# 2,3-Di­phenyl­male­imide 1-methyl­pyrrol­idin-2-one monosolvate

**DOI:** 10.1107/S1600536814002372

**Published:** 2014-02-08

**Authors:** Evgeny Bulatov, Dina Boyarskaya, Tatiana Chulkova, Matti Haukka

**Affiliations:** a Department of Chemistry, Saint Petersburg State University, Universitetsky Pr. 26, 198504 Stary Petergof, Russian Federation; b Department of Chemistry, University of Jyvaskyla, PO Box 35 FI-40014, Jyvaskyla, Finland

## Abstract

In the title compound, C_16_H_11_NO_2_·C_5_H_9_NO, the dihedral angles between the male­imide and phenyl rings are 34.7 (2) and 64.8 (2)°. In the crystal, the 2,3-di­phenyl­male­imide and 1-methyl­pyrrolidin-2-one mol­ecules form centrosymmetrical dimers *via* pairs of strong N—H⋯O hydrogen bonds and π–π stacking inter­actions between the two neighboring male­imide rings [centroid–centroid distance = 3.495 (2) Å]. The dimers are further linked by weak C—H⋯O and C—H⋯π hydrogen bonds into a three-dimensional framework.

## Related literature   

For general background to male­imides, see: Yeh *et al.* (2004 ▶); Billiet *et al.* (2011 ▶); Zhu *et al.* (2012 ▶); Parsons & Du Bois (2013 ▶). For the crystal structures of related compounds, see: Zhang *et al.* (2004 ▶); Mitzi & Afzali (2007 ▶).
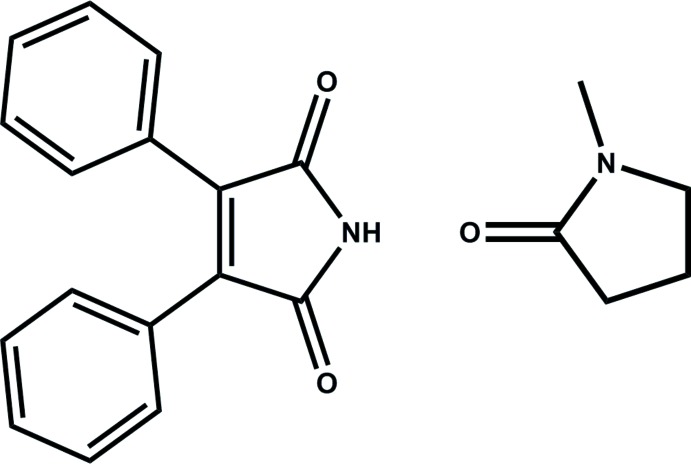


## Experimental   

### 

#### Crystal data  

C_16_H_11_NO_2_·C_5_H_9_NO
*M* *_r_* = 348.39
Monoclinic, 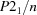

*a* = 13.1962 (3) Å
*b* = 10.0002 (2) Å
*c* = 13.5600 (3) Å
β = 100.469 (3)°
*V* = 1759.65 (7) Å^3^
*Z* = 4
Mo *K*α radiation
μ = 0.09 mm^−1^
*T* = 170 K
0.54 × 0.40 × 0.24 mm

#### Data collection  

Agilent SuperNova (Single source at offset, Eos) diffractometer
Absorption correction: multi-scan (*CrysAlis PRO*, Agilent, 2013 ▶) *T*_min_ = 0.815, *T*_max_ = 1.000
22206 measured reflections
8818 independent reflections
5708 reflections with *I* > 2σ(*I*)
*R*_int_ = 0.028

#### Refinement  

*R*[*F* ^2^ > 2σ(*F* ^2^)] = 0.064
*wR*(*F* ^2^) = 0.204
*S* = 1.03
8818 reflections
236 parameters
H-atom parameters constrained
Δρ_max_ = 0.63 e Å^−3^
Δρ_min_ = −0.30 e Å^−3^

### 

Data collection: *CrysAlis PRO* (Agilent, 2013 ▶); cell refinement: *CrysAlis PRO*; data reduction: *CrysAlis PRO*; program(s) used to solve structure: *SHELXTL* (Sheldrick, 2008 ▶); program(s) used to refine structure: *SHELXTL*; molecular graphics: *CrystalMaker* (CrystalMaker, 2011 ▶); software used to prepare material for publication: *OLEX2* (Dolomanov *et al.*, 2009 ▶) and *SHELXLE* (Hübschle *et al.*, 2011 ▶).

## Supplementary Material

Crystal structure: contains datablock(s) I, global. DOI: 10.1107/S1600536814002372/kq2011sup1.cif

Structure factors: contains datablock(s) I. DOI: 10.1107/S1600536814002372/kq2011Isup2.hkl

Supporting information file. DOI: 10.1107/S1600536814002372/kq2011Isup3.cml

CCDC reference: 978501

Additional supporting information:  crystallographic information; 3D view; checkCIF report

## Figures and Tables

**Table 1:** Hydrogen-bond geometry (Å, °) *Cg*2 and *Cg*3 are the centroids of the C3–C8 and C10–C15 rings, respectively.

*D*—H⋯*A*	*D*—H	H⋯*A*	*D*⋯*A*	*D*—H⋯*A*
N1—H1⋯O3^i^	0.88	1.95	2.7800 (15)	156
C5—H5⋯O1^ii^	0.95	2.41	3.3639 (17)	179
C21—H21*A*⋯O1^iii^	0.98	2.59	3.498 (2)	154
C21—H21*B*⋯O1^iv^	0.98	2.73	3.436 (2)	129
C15—H15⋯*Cg*2^v^	0.95	2.96	3.8081 (14)	149
C20—H20*A*⋯*Cg*3^iii^	0.99	2.91	3.6508 (18)	133

Symmetry codes: (i) 
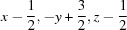
; (ii) 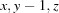; (iii) 
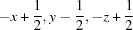
; (iv) 
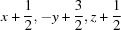
; (v) 
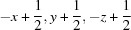
.

**Fig. 1.**
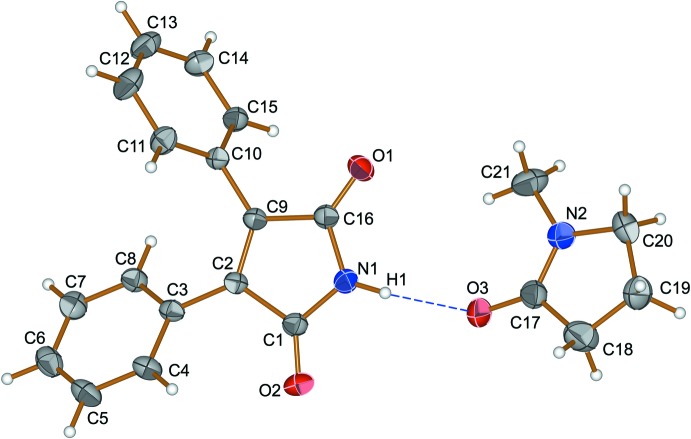
Molecular structure of the title compound with displacement ellipsoids drawn at the 50% probability level.

**Fig. 2.**
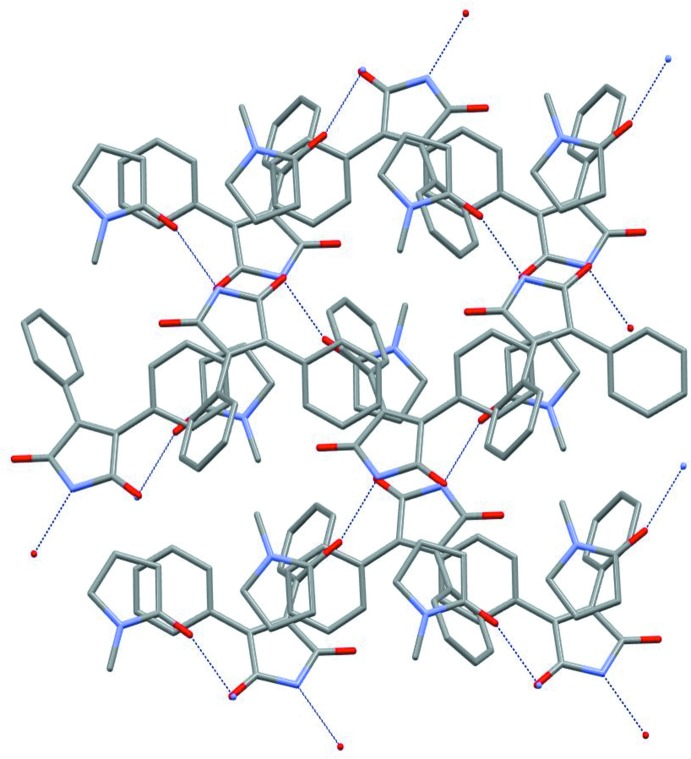
Crystal packing along the crystallographic a axis. All hydrogen atoms have been omitted for clarity.

## supplementary crystallographic information

### 1. Comment

Maleimide derivatives can be used as building blocks in the synthesis of a wide range of biologically active compounds (Parsons *et al.*, 2013), polymeric materials (Billiet *et al.*, 2011), nanoparticles (Zhu *et al.*, 2012), *etc*.

The present work describes the synthesis and crystal structure of 2,3-diphenylmaleimide 1-methylpyrrolidin-2-one monosolvate, C_16_H_11_NO_2_^.^C_5_H_9_NO (Fig. 1). The bond lengths and angles within the 2,3-diphenylmaleimide molecule (Table 1) are in a good agreement with those found in the related compounds (Zhang *et al.* (2004); Mitzi *et al.* (2007)). The dihedral angles between the maleimide and phenyl rings are 34.7 (2)° and 64.8 (2)°. In the crystal, the 2,3-diphenylmaleimide and 1-methylpyrrolidin-2-one molecules form centrosymmetrical dimeric associates *via* strong N—H···O hydrogen bonds (Table 2) and π-π stacking interactions between the two neighboring maleimide rings (the centroid-centroid distance is 3.495 (2) Å). Further the associates are linked by weak C—H···O (Table 2) and C—H···π hydrogen bonds into three-dimensional framework (Fig. 2).

### 2. Experimental

3,4-Diphenylpyrrol-2,5-diimine (0.810 mmol, 0.20 g) was hydrolyzed in 80% aqueous methanol (10 mL) for 24 h at room temperature. The yellow solid was obtained from the reaction mixture. The crystals of the title compound suitable for single crystal X-ray diffraction were obtained by recrystallization from 1-methylpyrrolidin-2-one.

### 3. Refinement

Structural refinement was carried out using *SHELXTL* (Sheldrick, 2008) with the *Olex2* (Dolomanov *et al.*, 2009) and *SHELXLE* (Hübschle *et al.*, 2011) graphical user interfaces. All hydrogen atoms were positioned geometrically and constrained to ride on their parent atoms, with C—H = 0.95–0.98 Å, N—H = 0.88 Å and U_iso_ = 1.2–1.5 U_eq_ (parent atom).

### Crystal data

**Table:**

C_16_H_11_NO_2_·C_5_H_9_NO	*F*(000) = 736
*M**_r_* = 348.39	*D*_x_ = 1.315 Mg m^−^^3^
Monoclinic, *P*2_1_/*n*	Mo *K*α radiation, λ = 0.71073 Å
*a* = 13.1962 (3) Å	Cell parameters from 5604 reflections
*b* = 10.0002 (2) Å	θ = 3.7–36.7°
*c* = 13.5600 (3) Å	µ = 0.09 mm^−^^1^
β = 100.469 (3)°	*T* = 170 K
*V* = 1759.65 (7) Å^3^	Block, colourless
*Z* = 4	0.54 × 0.40 × 0.24 mm

### Data collection

**Table:**

Agilent SuperNova (Single source at offset, Eos) diffractometer	8818 independent reflections
Radiation source: SuperNova (Mo) X-ray Source	5708 reflections with *I* > 2σ(*I*)
Mirror monochromator	*R*_int_ = 0.028
Detector resolution: 16.0107 pixels mm^-1^	θ_max_ = 37.5°, θ_min_ = 3.1°
φ scans and ω scans with κ offset	*h* = −22→22
Absorption correction: multi-scan (*CrysAlis PRO*, Agilent, 2013)	*k* = −13→16
*T*_min_ = 0.815, *T*_max_ = 1.000	*l* = −22→23
22206 measured reflections	

### Refinement

**Table:**

Refinement on *F*^2^	0 restraints
Least-squares matrix: full	Hydrogen site location: inferred from neighbouring sites
*R*[*F*^2^ > 2σ(*F*^2^)] = 0.064	H-atom parameters constrained
*wR*(*F*^2^) = 0.204	*w* = 1/[σ^2^(*F*_o_^2^) + (0.0916*P*)^2^ + 0.4242*P*] where *P* = (*F*_o_^2^ + 2*F*_c_^2^)/3
*S* = 1.03	(Δ/σ)_max_ < 0.001
8818 reflections	Δρ_max_ = 0.63 e Å^−^^3^
236 parameters	Δρ_min_ = −0.30 e Å^−^^3^

### Special details

**Table:**

Experimental. Absorption correction: *CrysAlis PRO* (Agilent, 2013); Empirical absorption correction using spherical harmonics, implemented in SCALE3 ABSPACK scaling algorithm.
Geometry. All e.s.d.'s (except the e.s.d. in the dihedral angle between two l.s. planes) are estimated using the full covariance matrix. The cell e.s.d.'s are taken into account individually in the estimation of e.s.d.'s in distances, angles and torsion angles; correlations between e.s.d.'s in cell parameters are only used when they are defined by crystal symmetry. An approximate (isotropic) treatment of cell e.s.d.'s is used for estimating e.s.d.'s involving l.s. planes.

### Fractional atomic coordinates and isotropic or equivalent isotropic displacement parameters (Å^2^)

**Table:**

	*x*	*y*	*z*	*U*_iso_*/*U*_eq_	
O1	0.06128 (8)	0.79304 (9)	0.09092 (7)	0.0333 (2)	
O2	0.16449 (8)	0.38928 (10)	−0.01022 (8)	0.0365 (2)	
N1	0.10893 (8)	0.60420 (10)	0.01257 (7)	0.0272 (2)	
H1	0.1127	0.6383	−0.0465	0.033*	
C1	0.13055 (9)	0.47257 (12)	0.04002 (9)	0.0260 (2)	
C2	0.10664 (8)	0.45632 (11)	0.14421 (8)	0.0230 (2)	
C3	0.12077 (8)	0.33036 (11)	0.20052 (9)	0.0240 (2)	
C4	0.09962 (10)	0.20708 (12)	0.15227 (10)	0.0300 (2)	
H4	0.0764	0.2042	0.0818	0.036*	
C5	0.11244 (11)	0.08926 (13)	0.20683 (12)	0.0366 (3)	
H5	0.0982	0.0059	0.1736	0.044*	
C6	0.14610 (11)	0.09271 (14)	0.31006 (12)	0.0370 (3)	
H6	0.1540	0.0118	0.3473	0.044*	
C7	0.16818 (10)	0.21392 (14)	0.35876 (11)	0.0334 (3)	
H7	0.1918	0.2160	0.4292	0.040*	
C8	0.15569 (9)	0.33222 (13)	0.30448 (9)	0.0275 (2)	
H8	0.1709	0.4152	0.3381	0.033*	
C9	0.07716 (8)	0.57673 (11)	0.17357 (8)	0.02304 (19)	
C10	0.04640 (8)	0.61711 (11)	0.26815 (8)	0.0232 (2)	
C11	−0.04123 (10)	0.56355 (15)	0.29686 (10)	0.0324 (3)	
H11	−0.0829	0.5013	0.2547	0.039*	
C12	−0.06732 (11)	0.60162 (19)	0.38744 (12)	0.0418 (3)	
H12	−0.1276	0.5662	0.4067	0.050*	
C13	−0.00602 (12)	0.69086 (17)	0.44990 (11)	0.0406 (3)	
H13	−0.0241	0.7158	0.5121	0.049*	
C14	0.08136 (12)	0.74379 (15)	0.42201 (10)	0.0362 (3)	
H14	0.1237	0.8042	0.4653	0.043*	
C15	0.10711 (10)	0.70841 (13)	0.33056 (9)	0.0290 (2)	
H15	0.1661	0.7465	0.3105	0.035*	
C16	0.08056 (9)	0.67456 (12)	0.09100 (9)	0.0254 (2)	
O3	0.67483 (9)	0.76077 (11)	0.35649 (9)	0.0429 (3)	
N2	0.65279 (9)	0.53588 (12)	0.32844 (9)	0.0348 (2)	
C17	0.66039 (10)	0.66246 (14)	0.30065 (11)	0.0333 (3)	
C18	0.64860 (19)	0.66600 (18)	0.18720 (12)	0.0554 (5)	
H18A	0.5915	0.7263	0.1580	0.066*	
H18B	0.7129	0.6983	0.1672	0.066*	
C19	0.62610 (16)	0.52685 (18)	0.15209 (12)	0.0483 (4)	
H19A	0.6767	0.4966	0.1112	0.058*	
H19B	0.5562	0.5208	0.1109	0.058*	
C20	0.63358 (14)	0.44090 (16)	0.24659 (13)	0.0447 (4)	
H20A	0.5685	0.3917	0.2467	0.054*	
H20B	0.6908	0.3758	0.2514	0.054*	
C21	0.65804 (14)	0.4951 (2)	0.43134 (12)	0.0479 (4)	
H21A	0.5906	0.4613	0.4404	0.072*	
H21B	0.6773	0.5719	0.4757	0.072*	
H21C	0.7098	0.4244	0.4478	0.072*	

### Atomic displacement parameters (Å^2^)

**Table:**

	*U*^11^	*U*^22^	*U*^33^	*U*^12^	*U*^13^	*U*^23^
O1	0.0447 (5)	0.0228 (4)	0.0320 (4)	0.0014 (4)	0.0059 (4)	0.0003 (3)
O2	0.0443 (5)	0.0350 (5)	0.0337 (5)	0.0028 (4)	0.0161 (4)	−0.0076 (4)
N1	0.0337 (5)	0.0265 (5)	0.0219 (4)	−0.0021 (4)	0.0062 (4)	−0.0016 (4)
C1	0.0263 (5)	0.0271 (5)	0.0247 (5)	−0.0012 (4)	0.0050 (4)	−0.0041 (4)
C2	0.0235 (4)	0.0221 (5)	0.0232 (4)	−0.0005 (3)	0.0039 (4)	−0.0028 (4)
C3	0.0219 (4)	0.0218 (4)	0.0283 (5)	0.0014 (4)	0.0046 (4)	−0.0026 (4)
C4	0.0304 (5)	0.0230 (5)	0.0355 (6)	0.0006 (4)	0.0028 (5)	−0.0054 (4)
C5	0.0329 (6)	0.0221 (5)	0.0536 (8)	0.0002 (4)	0.0049 (6)	−0.0022 (5)
C6	0.0316 (6)	0.0278 (6)	0.0515 (8)	0.0042 (5)	0.0074 (6)	0.0094 (6)
C7	0.0300 (5)	0.0358 (6)	0.0341 (6)	0.0071 (5)	0.0047 (5)	0.0060 (5)
C8	0.0277 (5)	0.0254 (5)	0.0292 (5)	0.0035 (4)	0.0040 (4)	−0.0002 (4)
C9	0.0242 (4)	0.0219 (4)	0.0230 (4)	−0.0008 (4)	0.0043 (4)	−0.0018 (4)
C10	0.0249 (4)	0.0219 (4)	0.0234 (4)	0.0016 (4)	0.0054 (4)	−0.0007 (4)
C11	0.0268 (5)	0.0382 (7)	0.0331 (6)	−0.0036 (5)	0.0077 (4)	−0.0008 (5)
C12	0.0332 (6)	0.0589 (10)	0.0371 (7)	−0.0004 (6)	0.0163 (5)	0.0034 (7)
C13	0.0460 (8)	0.0513 (9)	0.0275 (6)	0.0078 (6)	0.0148 (6)	−0.0004 (6)
C14	0.0479 (7)	0.0354 (7)	0.0258 (5)	−0.0018 (6)	0.0083 (5)	−0.0061 (5)
C15	0.0345 (6)	0.0270 (5)	0.0264 (5)	−0.0047 (4)	0.0081 (4)	−0.0038 (4)
C16	0.0275 (5)	0.0244 (5)	0.0239 (5)	−0.0018 (4)	0.0036 (4)	−0.0020 (4)
O3	0.0568 (6)	0.0368 (5)	0.0397 (5)	−0.0118 (5)	0.0214 (5)	−0.0127 (4)
N2	0.0366 (5)	0.0342 (6)	0.0347 (6)	−0.0033 (4)	0.0091 (4)	−0.0007 (5)
C17	0.0317 (5)	0.0324 (6)	0.0368 (6)	−0.0018 (5)	0.0085 (5)	−0.0040 (5)
C18	0.0896 (14)	0.0413 (9)	0.0326 (7)	−0.0106 (9)	0.0043 (8)	0.0021 (6)
C19	0.0630 (10)	0.0473 (9)	0.0359 (7)	−0.0088 (8)	0.0126 (7)	−0.0103 (7)
C20	0.0566 (9)	0.0310 (7)	0.0478 (8)	−0.0027 (6)	0.0134 (7)	−0.0093 (6)
C21	0.0487 (8)	0.0598 (10)	0.0359 (7)	−0.0088 (8)	0.0098 (6)	0.0105 (7)

### Geometric parameters (Å, º)

**Table:**

O1—C16	1.2118 (15)	C11—H11	0.9500
O2—C1	1.2121 (15)	C12—C13	1.385 (2)
N1—C16	1.3824 (15)	C12—H12	0.9500
N1—C1	1.3835 (16)	C13—C14	1.383 (2)
N1—H1	0.8800	C13—H13	0.9500
C1—C2	1.5113 (16)	C14—C15	1.3898 (18)
C2—C9	1.3476 (16)	C14—H14	0.9500
C2—C3	1.4672 (16)	C15—H15	0.9500
C3—C4	1.3998 (16)	O3—C17	1.2344 (17)
C3—C8	1.4013 (17)	N2—C17	1.3296 (19)
C4—C5	1.3851 (19)	N2—C21	1.4433 (19)
C4—H4	0.9500	N2—C20	1.448 (2)
C5—C6	1.390 (2)	C17—C18	1.518 (2)
C5—H5	0.9500	C18—C19	1.483 (2)
C6—C7	1.386 (2)	C18—H18A	0.9900
C6—H6	0.9500	C18—H18B	0.9900
C7—C8	1.3872 (18)	C19—C20	1.531 (2)
C7—H7	0.9500	C19—H19A	0.9900
C8—H8	0.9500	C19—H19B	0.9900
C9—C10	1.4703 (15)	C20—H20A	0.9900
C9—C16	1.4936 (16)	C20—H20B	0.9900
C10—C11	1.3926 (17)	C21—H21A	0.9800
C10—C15	1.3947 (16)	C21—H21B	0.9800
C11—C12	1.388 (2)	C21—H21C	0.9800
			
C16—N1—C1	110.41 (10)	C12—C13—H13	119.9
C16—N1—H1	124.8	C13—C14—C15	119.86 (13)
C1—N1—H1	124.8	C13—C14—H14	120.1
O2—C1—N1	125.57 (12)	C15—C14—H14	120.1
O2—C1—C2	127.79 (12)	C14—C15—C10	120.10 (12)
N1—C1—C2	106.62 (10)	C14—C15—H15	120.0
C9—C2—C3	129.02 (11)	C10—C15—H15	120.0
C9—C2—C1	107.49 (10)	O1—C16—N1	125.67 (12)
C3—C2—C1	123.40 (10)	O1—C16—C9	127.31 (11)
C4—C3—C8	118.88 (11)	N1—C16—C9	107.01 (10)
C4—C3—C2	121.14 (11)	C17—N2—C21	123.38 (14)
C8—C3—C2	119.97 (10)	C17—N2—C20	114.78 (13)
C5—C4—C3	120.32 (13)	C21—N2—C20	121.78 (14)
C5—C4—H4	119.8	O3—C17—N2	126.55 (14)
C3—C4—H4	119.8	O3—C17—C18	125.38 (14)
C4—C5—C6	120.16 (13)	N2—C17—C18	108.07 (13)
C4—C5—H5	119.9	C19—C18—C17	106.32 (14)
C6—C5—H5	119.9	C19—C18—H18A	110.5
C7—C6—C5	120.18 (13)	C17—C18—H18A	110.5
C7—C6—H6	119.9	C19—C18—H18B	110.5
C5—C6—H6	119.9	C17—C18—H18B	110.5
C6—C7—C8	119.91 (13)	H18A—C18—H18B	108.7
C6—C7—H7	120.0	C18—C19—C20	106.22 (13)
C8—C7—H7	120.0	C18—C19—H19A	110.5
C7—C8—C3	120.54 (12)	C20—C19—H19A	110.5
C7—C8—H8	119.7	C18—C19—H19B	110.5
C3—C8—H8	119.7	C20—C19—H19B	110.5
C2—C9—C10	130.04 (11)	H19A—C19—H19B	108.7
C2—C9—C16	108.31 (10)	N2—C20—C19	104.41 (13)
C10—C9—C16	121.64 (10)	N2—C20—H20A	110.9
C11—C10—C15	119.78 (11)	C19—C20—H20A	110.9
C11—C10—C9	120.87 (11)	N2—C20—H20B	110.9
C15—C10—C9	119.33 (10)	C19—C20—H20B	110.9
C12—C11—C10	119.61 (13)	H20A—C20—H20B	108.9
C12—C11—H11	120.2	N2—C21—H21A	109.5
C10—C11—H11	120.2	N2—C21—H21B	109.5
C13—C12—C11	120.44 (13)	H21A—C21—H21B	109.5
C13—C12—H12	119.8	N2—C21—H21C	109.5
C11—C12—H12	119.8	H21A—C21—H21C	109.5
C14—C13—C12	120.19 (13)	H21B—C21—H21C	109.5
C14—C13—H13	119.9		

### Hydrogen-bond geometry (Å, º)

Cg2 and Cg3 are the centroids of the C3–C8 and C10–C15 rings, respectively.

**Table:**

*D*—H···*A*	*D*—H	H···*A*	*D*···*A*	*D*—H···*A*
N1—H1···O3^i^	0.88	1.95	2.7800 (15)	156
C5—H5···O1^ii^	0.95	2.41	3.3639 (17)	179
C21—H21*A*···O1^iii^	0.98	2.59	3.498 (2)	154
C21—H21*B*···O1^iv^	0.98	2.73	3.436 (2)	129
C15—H15···*Cg*2^v^	0.95	2.96	3.8081 (14)	149
C20—H20*A*···*Cg*3^iii^	0.99	2.91	3.6508 (18)	133

Symmetry codes: (i) *x*−1/2, −*y*+3/2, *z*−1/2; (ii) *x*, *y*−1, *z*; (iii) −*x*+1/2, *y*−1/2, −*z*+1/2; (iv) *x*+1/2, −*y*+3/2, *z*+1/2; (v) −*x*+1/2, *y*+1/2, −*z*+1/2.
